# Systematic Nonenzymatic Glucose Detection Using Hydrogel-Protected Ag–Cu/MWCNTs Nanocomposites

**DOI:** 10.1021/acsomega.5c06932

**Published:** 2025-10-10

**Authors:** Jian-Kai Huang, Sin-Yu Chen, Meng-Chang Lin

**Affiliations:** Department of Materials Science and Engineering, 34916 National Chung Hsing University, Taichung 40227, Taiwan, ROC

## Abstract

The growing need for reliable and selective glucose monitoring has accelerated interest in nonenzymatic electrochemical sensors. In this work, we introduce a high-performance sensor based on silver–copper (Ag–Cu) alloy nanoparticles uniformly anchored onto multiwalled carbon nanotubes (MWCNTs), further enhanced with a polymeric protective layer composed of polyvinylpyrrolidone (PVP) and poly­(vinyl alcohol) (PVA). The materials were synthesized via a hydrothermal process, and the atomic ratio of Ag/Cu was precisely tuned. Among all compositions, the Ag_5_–Cu_5_ configuration exhibited the highest electrocatalytic activity, attributed to a strong bimetallic synergistic effect and the excellent conductivity of the MWCNTs framework. Cyclic voltammetry and amperometry measurements confirmed that the sensor achieved a sensitivity of 237.2 μA·mM^–1^·cm^–2^, a detection limit of 1.2 μM, and a linear response range spanning from 0.5 to 10 mM. The PVP/PVA hydrogel coating improved selectivity by acting as a molecular sieve, excluding interfering species like uric acid (UA) and ascorbic acid (AA) through electrostatic repulsion while allowing neutral glucose to diffuse freely. The integration of metal alloy nanostructures with hydrogel interface engineering offers a robust strategy for real-time glucose sensing in complex biofluids such as blood and sweat, advancing the development of next-generation nonenzymatic glucose sensors.

## Graphical abstract

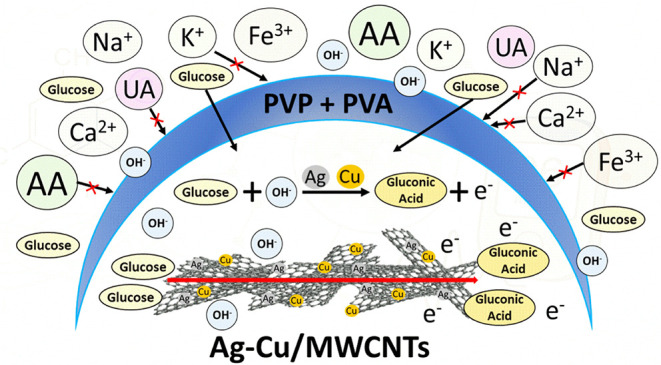

## 1 Introduction

In recent years, the number of individuals diagnosed with diabetes has been steadily increasing, making it one of the top ten leading causes of death globally.^1^ The primary cause of diabetes is abnormal insulin secretion,^2,3^ which prevents glucose in the bloodstream from being converted into energy needed by the body, resulting in elevated blood glucose levels. According to predictions by the International Diabetes Federation (IDF) and the World Health Organization (WHO), the global diabetic population is expected to rise from 135 million in 1995 to 643 million in 2030. By 2045, it is estimated that one in every eight adults, or approximately 783 million people, will be affected by diabetes.^4^ These alarming statistics highlight the urgent need for rapid, accurate, and affordable glucose monitoring technologies.

Among glucose-sensing techniques,^5–9^ electrochemical^5^ and optical^6^ detection mechanisms are the most common. Since Clark and Lyons developed the first enzymatic electrochemical glucose electrode in 1962,^10^ enzymatic electrochemical glucose sensors have been widely studied and utilized due to their miniaturization, simplicity, high sensitivity, and low cost. The development of glucose sensors^11,12^ can be categorized into first-, second-, and third-generation enzymatic glucose sensors and fourth-generation glucose sensors (i.e., nonenzymatic glucose sensors). The first-generation sensors rely on immobilizing glucose oxidase (GOx) on the electrode surface, with their oxygen dependence limiting applications in hypoxic blood environments.^13,14^ On the other hand, second-generation sensors use redox mediators^15^ to interact with GOx, but they exhibit lower sensitivity and accuracy. The following third-generation sensors address these limitations, although their performance is still susceptible to variations in pH and temperature.^16,17^ Recently, nonenzymatic glucose sensors have emerged, eliminating the need for GOx and enabling direct glucose oxidation on the electrode surface, thereby offering superior sensitivity, selectivity, and stability.^18–21^

Fourth-generation glucose sensors typically incorporate nanostructured metals as catalytic and conductive materials,^22–27^ while carbon-based or flexible substrates provide mechanical support.^28–31^ Common catalytic nanomaterials include Au,^32,33^ Ag,^34,35^ Pt,^36,37^ Cu,^38,39^ Co,^40,41^ and Ni.^42,43^ Among them, Ag and Cu stand out due to their high electrical conductivity, strong catalytic activity, affordability, and large surface area.^44–47^ As for support materials, carbon-based materials offer high conductivity, biocompatibility, and chemical stability. Notably, multiwalled carbon nanotubes (MWCNTs) provide an ideal balance between cost-effectiveness and performance.^48–51^ In 2015, Li’s group reported the successful preparation of Cu–Ag using natural leaves as a reducing agent and showed excellent sensitivity and electrochemical properties in glucose detection under alkaline conditions (0.1 M NaOH).^52^ Compared with the research of Li’s team, we carefully studied the differences between the various proportions of silver–copper alloys and proposed the use of hydrogel layers for protection against the influence of interfering substances. On this basis, we used a hydrothermal synthesis method to evenly anchor Ag–Cu nanoparticles on acid-treated MWCNTs to form Ag–Cu/MWCNTs composites. This composite material is designed to enhance electron transfer efficiency and catalytic activity under near-neutral pH conditions, closely mimicking the human physiological environment, making it a promising candidate for nonenzymatic glucose detection.

Although fourth-generation glucose sensors eliminate enzymatic components and thus reduce biological variability, several endogenous substances, such as metal ions^53^ uric acid, and ascorbic acid^54,55^ can still interfere with glucose detection by competing for active sites or altering local electrochemical conditions. These interferences pose significant challenges for practical blood sample analysis. To address this, polymeric protective films, such as poly­(vinyl alcohol) (PVA) and polyvinylpyrrolidone (PVP), have been employed to act as molecular sieves, allowing small glucose molecules to diffuse through while rejecting larger interfering species.^56,57^ In this work, we further explore the synergistic effect of integrating Ag–Cu/MWCNTs with selectively permeable hydrogel films. By coating the electrode surface with a carefully chosen protective layer, we aim to enhance the detection specificity while preserving the catalytic efficiency. This dual-layer design serves as a barrier against interfering compounds and stabilizes the electrochemical interface, ensuring consistent glucose sensing in complex biological environments.

In this study, we propose a nonenzymatic glucose sensor based on an Ag–Cu/MWCNTs composite with a functional hydrogel protective layer. Ag–Cu/MWCNTs are synthesized by a hydrothermal method and optimized to provide high electrocatalytic activity at near-neutral pH. Meanwhile, we evaluate the effectiveness of different concentrations of polymer coatings (e.g., a mixture of PVP and PVA) in excluding common interfering substances in blood (e.g., ascorbic acid, uric acid, and metal ions). The main objectives of this study are to (1) investigate the electrochemical performance of Ag–Cu/MWCNTs in glucose oxidation, (2) evaluate the interference-blocking ability of the hydrogel films, and (3) assess the combined effects of these materials on the selectivity, stability, and practical usability of the sensor. This work aims to provide comprehensive material-structure–property relationships to advance the development of nonenzymatic glucose sensors for practical biomedical applications.

## 2 Experimental Methods

### 2.1 Functionalization of Multiwalled Carbon Nanotubes (MWCNTs)

Functionalization of MWCNTs was carried out by introducing carboxyl groups onto MWCNTs via acid treatment with H_2_SO_4_ and HNO_3_.^58,59^ Two grams of multiwalled carbon nanotubes (MWCNTs; diameter: 10–30 nm, Conjutek, Taiwan) were weighed and placed in a PTFE-lined stainless steel hydrothermal autoclave. Subsequently, 30 mL of concentrated sulfuric acid (H_2_SO_4_, >95%, Fisher Scientific, USA) and 10 mL of nitric acid (HNO_3_, 70%, JT-Baker, USA) were sequentially added. The hydrothermal reaction was conducted at 150 °C for 24 h to enhance the dispersibility of the MWCNTs in aqueous media and to introduce carboxylic acid functional groups at defect sites.^60^ After being cooled to room temperature, the post-treated MWCNTs were repeatedly washed with deionized water via centrifugation until a neutral pH was achieved. The functionalized MWCNTs were then collected by vacuum filtration and dried at 40 °C.

### 2.2 Preparation of Ag–Cu/MWCNTs Nanocomposite

The Ag–Cu/MWCNTs nanocomposite was synthesized via a chemical reduction method. Typically, 400 mg of functionalized multiwalled carbon nanotubes (f-MWCNTs) were dispersed in 20 mL of deionized water by ultrasonication for 30 min to achieve a homogeneous suspension. Subsequently, 0.394 g of silver nitrate (AgNO_3_, 99.8%, Sigma-Aldrich, USA) and 0.628 g of copper­(II) sulfate (CuSO_4_, 97.5%, Showa, Japan) were dissolved in the f-MWCNTs suspension. To initiate the reduction, 1.69 g of glucose (99%, Acros Organics, Belgium) was added as a reducing agent.^61,62^ The mole ratio of Ag/Cu was maintained at 1:1. The resulting mixture was transferred into a PTFE–lined stainless steel autoclave and subjected to hydrothermal treatment at 200 °C for 24 h.

After cooling to room temperature, the Ag–Cu/MWCNTs nanocomposite was thoroughly washed with deionized water using vacuum filtration and then dried in an oven at 50 °C for approximately 24 h. Additionally, nanocomposites with varying Ag-to-metal mole ratios (1:9 and 9:1), as well as monometallic and Ag–Cu alloy systems, were synthesized using this method.

### 2.3 Preparation of Glucose Detection Electrode

A total of 0.3 g of Ag–Cu/MWCNTs nanocomposites was weighed and individually dispersed in 6.39 mL of *N*,*N*–dimethylformamide (DMF, 99.8%, JT-Baker, USA). The dispersions were subjected to ultrasonication for 30 min to ensure uniformity. Subsequently, 0.034 g of polyvinylidene fluoride (PVDF, >99%, Syensqo, Belgium) was added to each dispersion, followed by magnetic stirring at 40 °C for 3 h to obtain a homogeneous ink. Then, 3 μL of the resulting suspension was quantitatively deposited onto the surface of a glassy carbon electrode (GCE, 3 mm diameter) using a micropipette and dried in an oven at 40 °C for 15 min. After confirming that the nanomaterials on the glassy carbon electrode remained intact and fully dried, 10 μL of a polyvinylpyrrolidone (PVP, 58,000 MW, Thermo Scientific, USA)/poly­(vinyl alcohol) (PVA, 146,000 MW, Acros Organics, USA) protective layer (0.3, 3, 10, and 30 wt %) was carefully dropped onto the electrode surface using a micropipette. The electrode was then dried in an oven at 40 °C for 30 min.

### 2.4 Characterization

The morphology and elemental composition of the synthesized nanocomposites were analyzed by using scanning electron microscopy (SEM) and energy-dispersive X-ray spectroscopy (EDX) with a JSM-6700F instrument (JEOL, Japan). The crystallographic structure was investigated by X-ray diffraction (XRD) using a D8 Advance ECO diffractometer (Bruker, USA) equipped with Cu Kα radiation. Transmission electron microscopy (TEM) images were obtained by using a field emission electron microscope operating at 200 kV (JEM-2010, JEOL, Japan).

Electrochemical measurements were carried out in a conventional three-electrode setup using a potentiostat (SP-50e, Biologic Science Instruments, France) at room temperature. The working electrodes consisted of a bare glassy carbon electrode (GCE), or GCE modified with the Ag–Cu/MWCNTs nanocomposites or a PVP/PVA protective layer. A saturated Ag/AgCl electrode and a bare GCE served as the reference and counter electrodes, respectively.

The electrochemical activity of the modified electrodes was determined using cyclic voltammetry (CV) in aqueous glucose solutions (0–12 mM) with 0.1 M NaOH (≥98%, Sigma-Aldrich, USA). A series of CVs were recorded for the Ag–Cu/MWCNTs-modified GCE at various scan rates, with the potential window ranging from −0.2 to 0.7 V (vs Ag/AgCl). The nonenzymatic glucose oxidation in an alkaline medium is typically an irreversible process involving catalytic electrochemical oxidation reactions. The interference effects of metal chlorides (Na^+^, K^+^, Ca^2+^, and Fe^3+^), ascorbic acid (AA), and uric acid (UA) on the performance of the electrodes were also investigated.

## 3 Results and Discussion

### 3.1 SEM and EDX Analyses

The morphology of the Ag–Cu/MWCNTs nanocomposites was characterized by using scanning electron microscopy (SEM). Figure 1A–E shows the SEM micrographs of Ag/MWCNTs, Cu/MWCNTs, Ag_1_–Cu_9_/MWCNTs, Ag_5_–Cu_5_/MWCNTs, and Ag_9_–Cu_1_/MWCNTs nanocomposites, respectively. As observed, the synthesized nanocomposites exhibit an approximately spherical morphology with minimal agglomeration. SEM analysis further indicates that the particles are well-ordered and uniformly distributed across the MWCNTs support. SEM analysis has shown that the as-prepared nanocomposites are well-ordered and uniformly distributed. The elemental composition of the as-prepared Ag_5_–Cu_5_/MWCNTs nanocomposites was analyzed from EDX spectra, as shown in Figure S1. The figure shows that the as-prepared Ag_5_–Cu_5_/MWCNTs nanocomposite contained different elements like Ag, Cu, C, and Si. The percentage composition by atomic of Ag and Cu was found to be 2.05 and 1.93 at. %, respectively, for Ag_5_–Cu_5_/MWCNTs, while for Ag_9_–Cu_1_/MWCNTs the atomic percentage composition of Ag and Cu was found to be 0.53 and 0.56 at. %, respectively. In the case of Ag_1_–Cu_9_/MWCNTs, the composition of Ag and Cu by weight was found to be 1.30 and 0.08 at. %, respectively (Figure 1F). These values align well with the molar ratios used during synthesis. The deviation in the Ag_9_–Cu_1_ sample is attributed to the partial precipitation of Ag^+^ ions as Ag_2_SO_4_ due to the presence of SO_4_^2–^ from copper sulfate during hydrothermal synthesis. The presence of Si in the EDX spectrum is due to the use of glass fiber filter paper for vacuum filtration during the initial cleaning of the acidified MWCNTs and the subsequent Ag–Cu/MWCNTs composites preparation.

**Figure 1:**
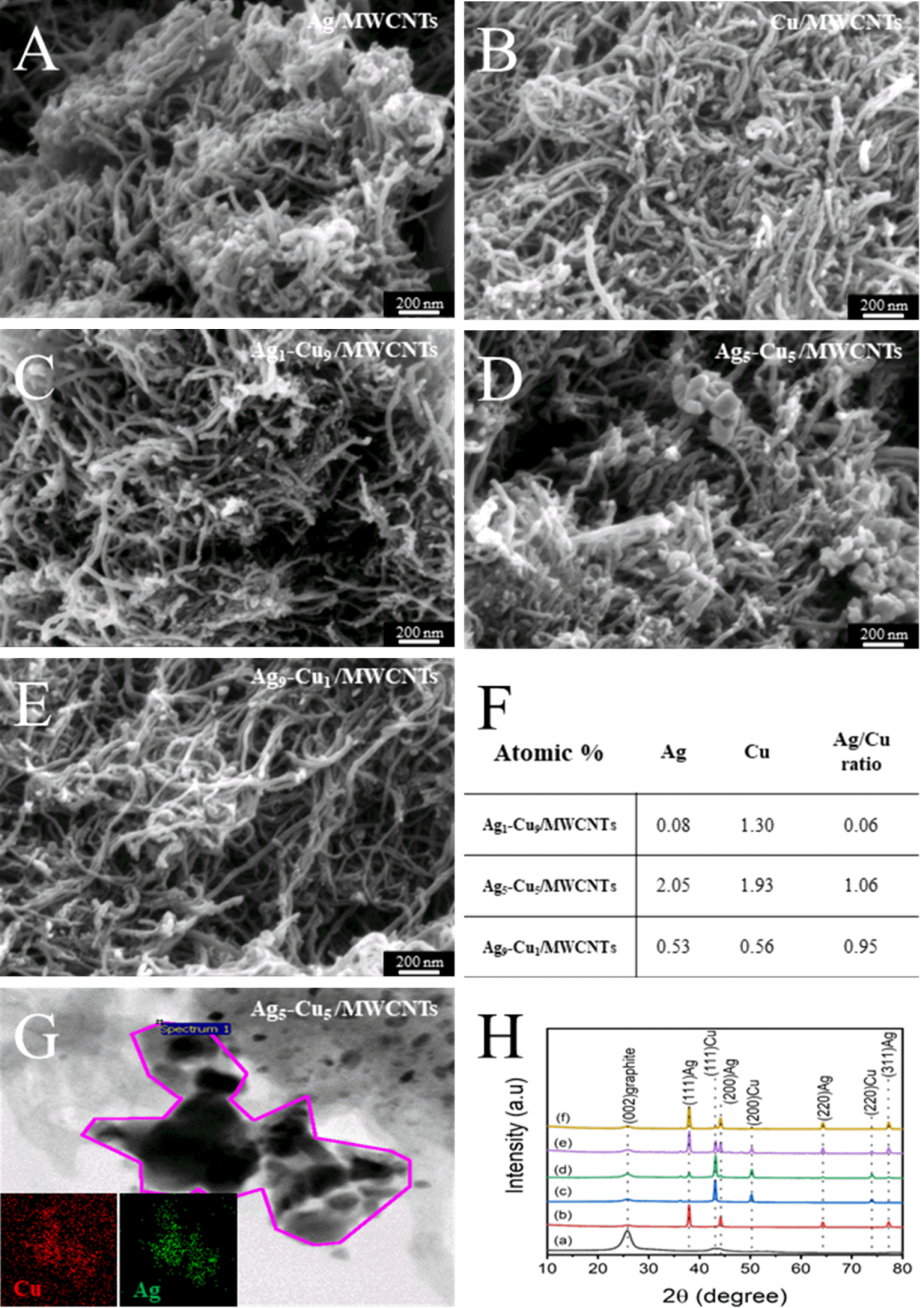
SEM micrographs of (A) Ag/MWCNTs, (B) Cu/MWCNTs, (C) Ag_1_–Cu_9_/MWCNTs, (D) Ag_5_–Cu_5_/MWCNTs, and (E) Ag_9_–Cu_1_/MWCNTs nanocomposites, showing uniformly dispersed metal nanoparticles on the MWCNT framework. (F) Atomic percentages and Ag/Cu ratios of nanocomposites with varying silver–copper compositions, based on EDX analysis. (G) TEM image and corresponding elemental mapping of Ag_5_–Cu_5_/MWCNTs, confirming the colocalized distribution of Ag and Cu. (H) XRD patterns of (a) MWCNTs, (b) Ag/MWCNTs, (c) Cu/MWCNTs, (d) Ag_1_–Cu_9_/MWCNTs, (e) Ag_5_–Cu_5_/MWCNTs, and (f) Ag_9_–Cu_1_/MWCNTs, indicating the crystalline phases of Ag, Cu, and the presence of MWCNTs.

Across all samples, the carbon content remains nearly constant, as the same amount of MWCNTs (400 mg) was used as the support. Silicon signals were consistently detected and attributed to contamination from the glass fiber filter paper used during filtration. Additionally, trace elements such as Fe, S, and Co were present across all samples, indicating impurities originating from the commercial MWCNTs.

### 3.2 TEM Analyses

The morphology of the nanocomposites prepared on MWCNTs (i.e., Ag_5_–Cu_5_/MWCNTs) was further confirmed by using transmission electron microscopy (TEM), as shown in Figure 1G. The TEM image reveals that the majority of the nanoparticles were uniformly deposited on the surface of the MWCNTs, indicating that the proposed synthesis method is effective in directing the growth of metal nanoparticles onto the MWCNTs matrix. Additionally, elemental mapping analysis (insets of Figure 1G) provides further evidence of the spatial distribution of the constituent elements. The red and green signals correspond to Cu and Ag, respectively, and their strong overlap in the mapped region confirms the homogeneous distribution of Ag and Cu within the nanocomposite, forming alloyed nanoparticles anchored on MWCNTs.

The bonding interaction between Ag–Cu and MWCNTs during nanocomposite formation can be interpreted through the coreduction mechanism. Silver ions (Ag^+^) are reduced earlier than copper ions (Cu^2+^) due to their higher standard reduction potential (0.799 V for Ag^+^/Ag^0^ vs 0.337 V for Cu^2+^/Cu^0^). Upon the introduction of glucose as a reducing agent, the difference in redox potential between Ag^+^/Ag^0^ and Cu^2+^/Cu^0^ decreases, but Ag^+^ still undergoes preferential reduction. Subsequently, Cu^2+^ is reduced onto the preformed Ag matrix, forming Ag–Cu solid solution nanoparticles, with small Cu atoms embedded in the silver lattice.^63,64^ Through glucose-mediated reduction, the carboxyl-functionalized MWCNTs (MWCNTs–COO^–^) interact with Ag^0^ and Cu^0^, resulting in the successful formation of Ag–Cu/MWCNTs nanocomposites.^65,66^

### 3.3 XRD Analyses

Figure 1H presents the XRD patterns of the synthesized nanocomposites. The strong diffraction peak observed at a 2-theta of 26° corresponds to the (002) plane of MWCNTs, as shown in pattern (a). Prominent peaks at 38.1°, 44.3°, 64.5°, and 77.5° are attributed to the (111), (200), (220), and (311) planes of metallic Ag (JCPDS card no. 03-0931), as observed in pattern (b). Additionally, the diffraction peaks located at 43.3°, 50.4°, and 74.1° correspond to the (111), (200), and (220) planes of metallic Cu (JCPDS card no. 02-1225), as seen in pattern (c). With increasing silver content, a noticeable decrease in the intensity of copper peaks was observed, along with the dominance of silver’s crystal structure, as evident in patterns (d–f). This trend was attributed to the homogeneous distribution of metallic Cu in a noncrystalline or monatomic form within the Ag lattice.^67^ These XRD results confirm the successful alloying of Ag and Cu, as well as their uniform dispersion on the surface of the MWCNTs.

The average crystallite size was further calculated using the Scherrer equation^68^ based on the Ag (111) diffraction peak. According to the experimental FWHM values, the Ag_1_–Cu_9_/MWCNTs, Ag_5_–Cu_5_/MWCNTs, and Ag_9_–Cu_1_/MWCNTs samples exhibited FWHM values of 0.41304, 0.33287, and 0.34674, respectively, corresponding to average crystallite sizes of 19.64, 24.36, and 23.42 nm. These results indicate that the 1:1 Ag-to-Cu mole ratio leads to the coarsest crystallites, suggesting that this composition is favorable for particle growth and crystallization. In contrast, a lower Ag content (Ag_1_–Cu_9_) results in significantly smaller grain sizes, possibly due to the difficulty in forming a stable alloy or the presence of heterogeneous interfacial forces.

Overall, Ag and Cu can effectively form Ag–Cu alloy nanoparticles via hydrothermal coreduction, achieving uniform dispersion on the MWCNTs surface (Figure 1). Due to the higher standard reduction potential of Ag^+^ (0.799 V) compared to Cu^2+^ (0.337 V), Ag^+^ is preferentially reduced to form Ag nuclei, followed by the reduction and deposition of Cu^2+^ onto the Ag surface, thereby forming the alloy structure. Moreover, glucose plays a critical role as a reducing agent, mitigating the potential difference between the two metals and facilitating a more coordinated reduction process.^63^ This alloying behavior and crystallite size control are crucial for subsequent applications in nonenzymatic glucose sensors, as the particle size and surface crystallinity directly influence electrocatalytic performance and stability. In the future, we will use other reducing agents, such as sodium borohydride,^69^ to study the influence of the morphology of the synthesized bimetallic alloys.

### 3.4 Electrochemical Characterization

#### 3.4.1 Electrochemical Activity Ag–Cu/MWCNTs toward Glucose

The electrocatalytic activity of those Ag–Cu/MWCNTs nanocomposites toward the oxidation of glucose was investigated by using cyclic voltammetry in 0.1 M NaOH at a scan rate of 50 mV/s (Figure 2). Upon scanning the potential in the range of −0.2 to 0.7 V (vs Ag/AgCl), no obvious redox peak was observed on bare GCE and MWCNTs-modified GCE for 0.5 mM glucose in 0.1 M NaOH (Figure 2A,B). These data show the passive nature of bare GCE and MWCNTs/GCE toward glucose oxidation in the potential range of −0.2 to 0.7 V against Ag/AgCl. However, a pair of well-defined redox peaks was observed at Ag/MWCNTs and Cu/MWCNTs electrodes (Figure 2C,D), which show better electrocatalytic activity toward glucose oxidation. Among all the mentioned electrodes, Ag_5_–Cu_5_/MWCNTs exhibited the best electrocatalytic reaction for glucose oxidation (Figure 2E). Clear anodic and cathodic peaks were recorded at 0.42 and 0.08 V (vs Ag/AgCl), respectively. This further increase in the anodic current was assigned to the presence of synergy between Ag and Cu atoms,^70^ as well as the larger surface area and greater number of catalytic sites provided by MWCNTs.

**Figure 2:**
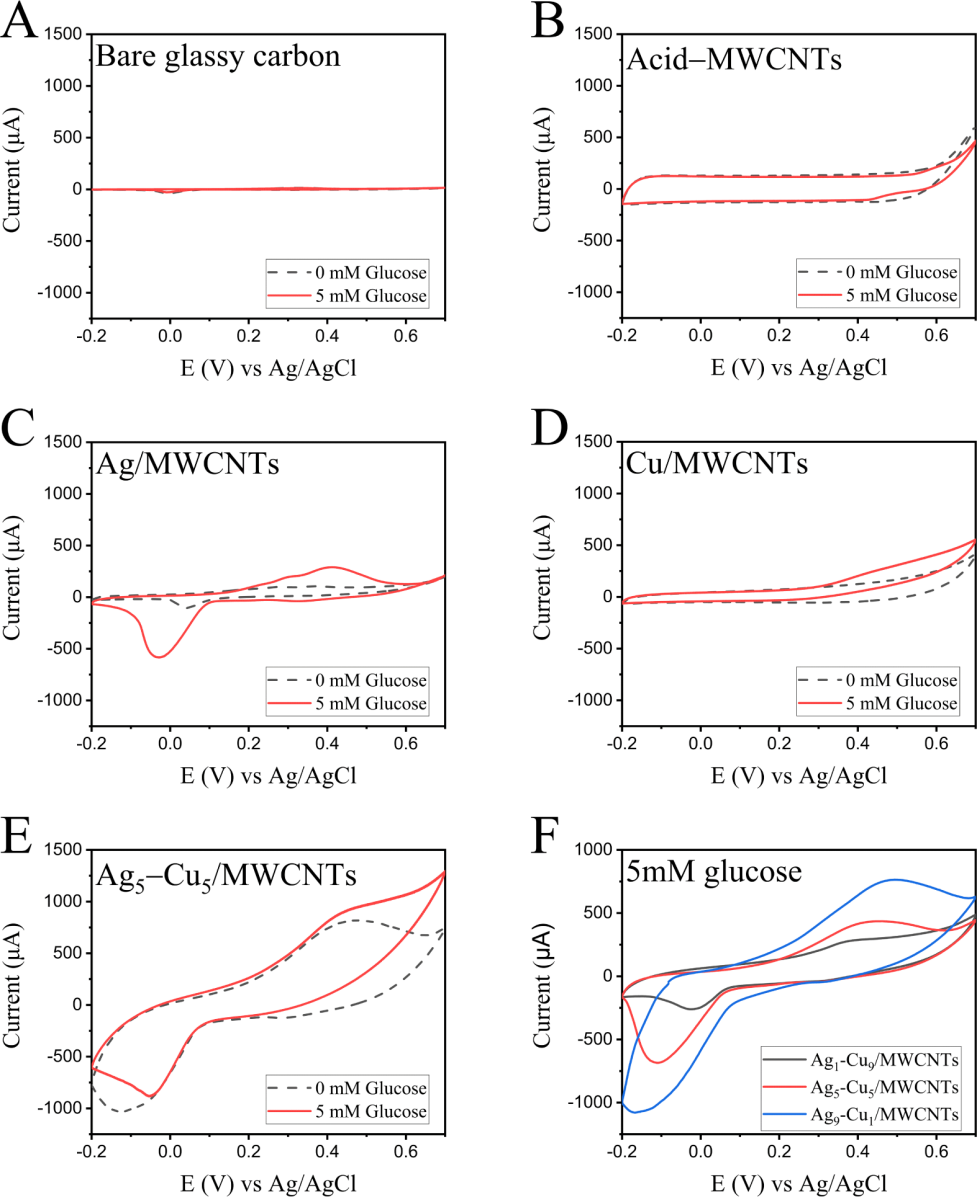
Cyclic voltammograms (CVs) recorded in 0.1 M NaOH solution at a scan rate of 50 mV/s for 0 and 5 mM glucose on: (A) bare glassy carbon electrode (GCE), (B) acid-treated MWCNTs electrode (Acid-MWCNTs), (C) Ag/MWCNTs electrode, (D) Cu/MWCNTs electrode, and (E) Ag_5_–Cu_5_/MWCNTs electrode. (F) CV curves of Ag–Cu/MWCNTs electrodes with different Ag/Cu ratios (Ag_1_–Cu_9_, Ag_5_–Cu_5_, and Ag_9_–Cu_1_) recorded in 5 mM glucose, illustrating the influence of metal composition on electrocatalytic performance toward glucose oxidation.

#### 3.4.2 Electrochemical Activity of Ag–Cu Alloys with Different Ratios toward Glucose

The electrocatalytic activity of the Ag_5_–Cu_5_/MWCNTs electrodes for glucose oxidation was studied by cyclic voltammetry in 0.1 M NaOH at a scan rate of 50 mV/s (Figure 2F). When scanning the potential range from −0.2 to 0.7 V (vs Ag/AgCl) in the presence of 2 mM glucose, it was observed that the redox peak current varied significantly with the Ag-to-Cu ratio. The maximum oxidation peak was observed for the Ag_9_–Cu_1_ composition, indicating that the glucose oxidation reaction is primarily governed by the Ag component. The oxidation peak appeared near 0.45 V, suggesting that this potential is optimal for subsequent amperometric measurements. Lu’s research group^71^ has demonstrated that nonenzymatic glucose sensors typically employ nanometals and metal oxides to directly catalyze glucose oxidation in strongly alkaline media, during which electrons are released. In such systems, cyclic voltammetry (CV) measurements usually reveal a distinct oxidation peak without a corresponding reduction peak, suggesting that the electrochemical oxidation of glucose is irreversible. However, in our study (Figure 2C,E,F), a clear reduction peak (at 0 to −0.15 V (vs Ag/AgCl)) was observed, which can be attributed to the electrochemical reduction of silver.^72^

It is well-known that the amperometric current response of a biosensor strongly depends on the applied potential.^73^ For better results, therefore, the amperometric glucose-sensing response of the Ag_5_–Cu_5_/MWCTs was tested at three different potentials of 0.40 (Figure 3A), 0.45 (Figure 3B), and 0.50 V (Figure 3C) vs Ag/AgCl under the conditions of constant stirring. Among these, 0.45 V provided a stable and high current response, while 0.50 V produced a higher but less stable signal. Therefore, 0.45 V was selected as the optimal working potential for further amperometric studies.

**Figure 3:**
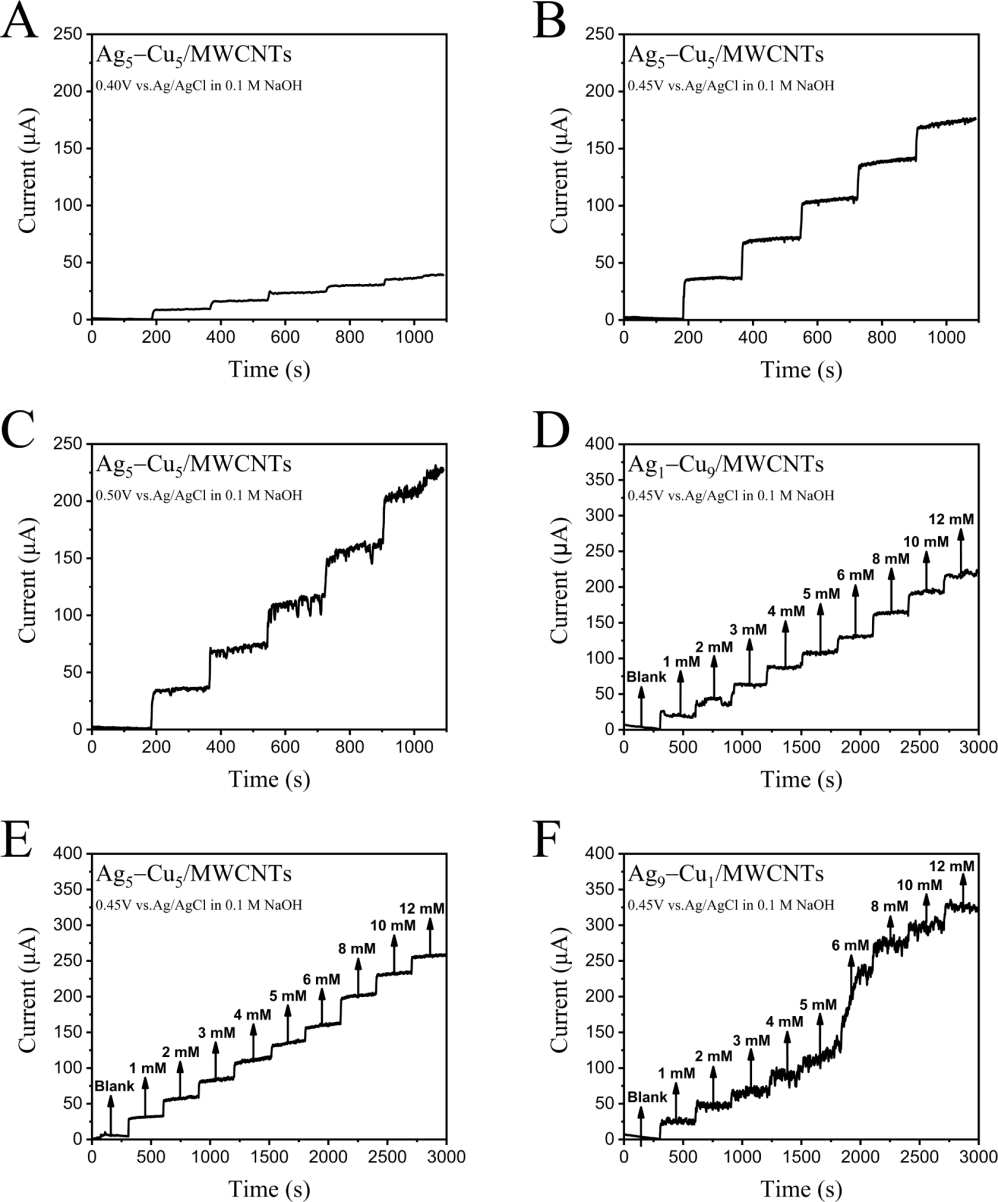
Amperometric responses recorded for glucose detection at different electrode materials and applied potentials. Current–time (i–t) responses of Ag_5_–Cu_5_/MWCNTs electrode at applied potentials of (A) 0.40 V, (B) 0.45 V, and (C) 0.50 V vs Ag/AgCl in 0.1 M NaOH, upon successive additions of glucose. Amperometric responses at 0.45 V vs Ag/AgCl for electrodes with different Ag/Cu ratios: (D) Ag_1_–Cu_9_/MWCNTs, (E) Ag_5_–Cu_5_/MWCNTs, and (F) Ag_9_–Cu_1_/MWCNTs, in response to successive additions of glucose from 1 to 12 mM. The arrows indicate the time points of glucose addition.

The three Ag–Cu alloys with different ratios were tested by amperometry, the glucose concentration was gradually increased, and the change of current at 0.45 V was observed (Figure 3D–F). It can be observed that the current is proportional to the glucose concentration within 1 to 12 mM. Among the three Ag–Cu ratios, it can be observed that the current of the Ag–Cu/MWCNTs electrode increases with the increase of the Ag ratio. In Ag_1_–Cu_9_/MWCNTs, it can be found that its current is slightly higher than that of Cu/MWCNTs because the active sites are increased after the addition of Ag modification. Although the ampere current is the largest in Ag_9_–Cu_1_/MWCNTs, the current stability is extremely poor (Figure 3F). Ag_5_–Cu_5_/MWCNTs has the advantage of high current over Ag_1_–Cu_9_ and current stability over Ag_9_–Cu_1_ (Figure 3D–F). Therefore, the Ag_5_–Cu_5_/MWCNTs electrode was selected to perform the following experiments.

#### 3.4.3 Effects of Concentration of Glucose and Scan Rate

The electrochemical response of Ag_5_–Cu_5_/MWCNTs toward glucose oxidation was investigated by using amperometric measurements with the consecutive addition of glucose into a 0.1 M NaOH supporting electrolyte at a scan rate of 50 mV/s. Given that the normal clinical range of blood glucose is approximately 3.9–5.5 mM, the glucose detection range was set between 0 and 10 mM. Under continuous stirring, glucose was added every 3 min: 0.5 mM increments were used for concentrations ranging from 0 to 5 mM, and 1 mM increments for the 5–10 mM range (Figure 4A). A strong linear relationship was observed between the current response and glucose concentration, with a correlation coefficient of *R* ^2^ = 0.99697 over the range of 0.5 to 10 mM (Figure 4B). Given that glucose concentrations in human sweat range from 0.06–0.2 mM, corresponding to approximately 3.3–17.3 mM in blood, the Ag_5_–Cu_5_/MWCNTs electrode sensor could, in the future, be applied for glucose detection in both blood and sweat.

**Figure 4:**
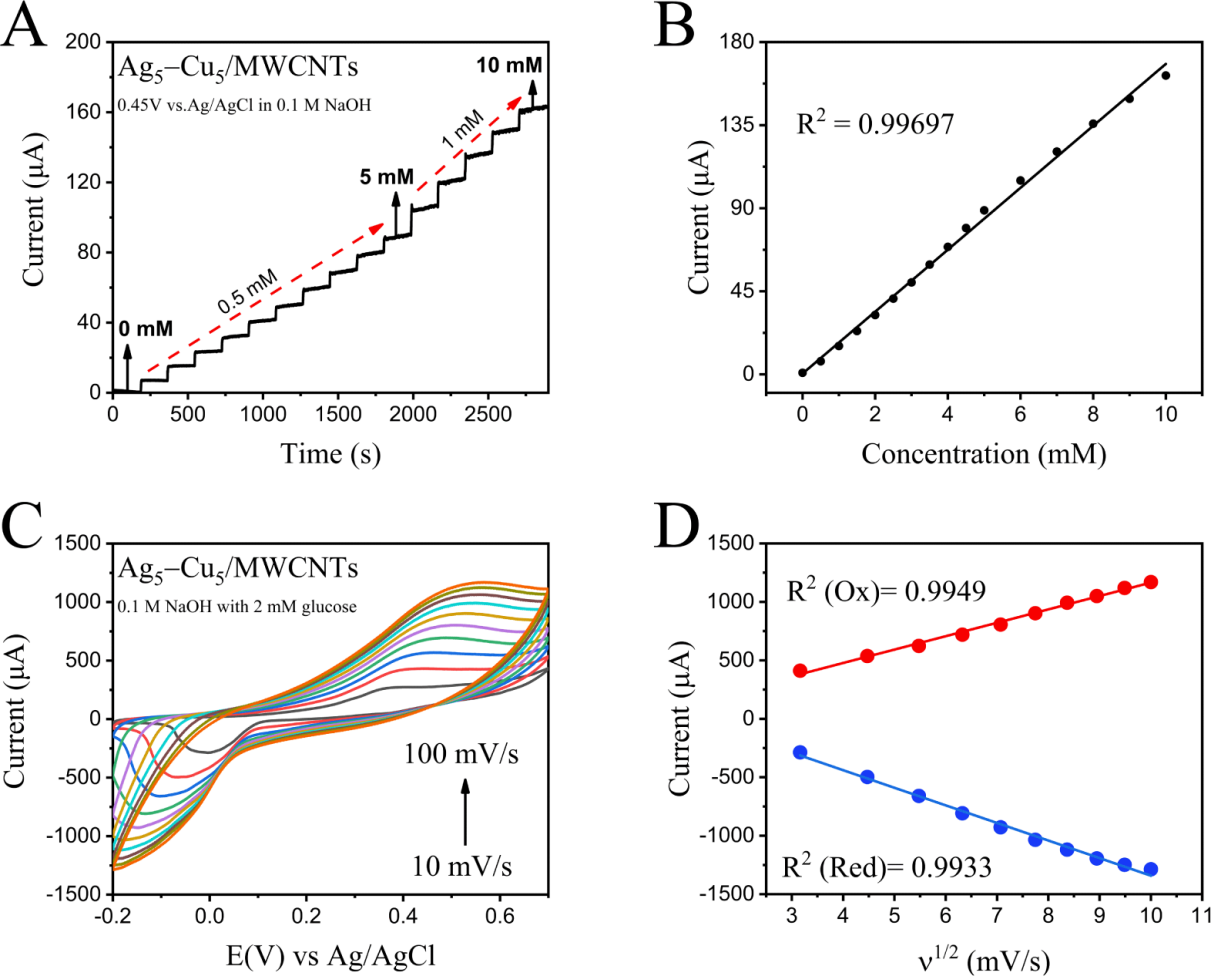
(A) Amperometric response of the Ag_5_–Cu_5_/MWCNTs electrode to successive additions of glucose (0–10 mM) at an applied potential of 0.45 V vs Ag/AgCl in 0.1 M NaOH, showing a stepwise increase in current with glucose concentration. (B) Corresponding calibration curve of current vs glucose concentration, exhibiting excellent linearity with a correlation coefficient of *R* ^2^ = 0.99697. (C) Cyclic voltammograms of Ag_5_–Cu_5_/MWCNTs recorded in 2 mM glucose at scan rates ranging from 10 to 100 mV/s, demonstrating scan rate-dependent electrochemical behavior. (D) Linear relationships between anodic and cathodic peak currents (*I*_p_) and the square root of scan rate (ν1/2), confirming diffusion-controlled kinetics. The correlation coefficients are *R* ^2^ = 0.9949 (oxidation) and *R* ^2^ = 0.9933 (reduction).

The scan rate effect can be used to collect the information about the kinetics of electrochemical reactions occurring on the electrode.^74^ The effect of scan rates on the CV of 2 mM glucose in 0.1 M NaOH was investigated at the modified electrode (Figure 4C). A linear relationship was observed between anodic and cathodic peak currents and with the square root of scan rates with a correlation coefficient (*R* ^2^) value of 0.9949 and 0.9933 for anodic and cathodic peaks, respectively (Figure 4D). The figure illustrates that the anodic peaks shift toward more positive potentials with increasing scan rates, indicating that the oxidation of glucose at the surface of the Ag–Cu/MWCNTs is a diffusion-controlled process. Furthermore, according to the Randles–Sevcik equation (*I*_p_ = 2.69 × 10^5^ n^3/2^ A D^1/2^ C ν^1/2^), a linear relationship between the anodic peak current (*I*_p_) and the square root of the scan rate (ν^1/2^) further confirms that the electrochemical oxidation of glucose on Ag_5_–Cu_5_/MWCNTs follows a diffusion-controlled mechanism. The amperometric response is linearly correlated with glucose concentration in the range of 0.05–10 mM (*R* ^2^ = 0.99697) (Figure 4B). The linear response corresponds to a sensitivity of 237.2 μA mM^–1^ cm^–2^. The detection limit of the Ag_5_–Cu_5_/MWCNTs electrode was calculated to be as low as 1.2 μM. It is worth mentioning that, similar to previously reported Cu–Ag/NF electrodes,^52,75^ the fabricated electrode exhibits electrocatalytic performance toward glucose detection in terms of sensitivity, detection limit, and linear range. Table 1 presents a comprehensive comparison of various nanomaterial-modified electrodes for nonenzymatic glucose sensing, focusing on key analytical parameters such as sensitivity, limit of detection (LOD), linear range, interference tolerance, and the presence of protective layers.^76–83^ The Ag_5_–Cu_5_/MWCNTs/GCE electrode developed in this work exhibits a sensitivity of 237.20 μA mM^–1^ cm^–2^, an LOD of 1.2 μM, and a linear detection range from 0.5 to 10.0 mM, positioning it competitively among recent advancements. Notably, while the Ag–Cu/NF electrode showed an exceptionally high sensitivity (7745.7 μA mM^–1^ cm^–2^) and a wide range, it lacked protective layering and had limited interference protection, only tolerating ascorbic acid (AA) and uric acid (UA). In contrast, the Ag_5_–Cu_5_/MWCNTs system in this study demonstrated broad interference resistance, effectively tolerating common interfering substances such as NaCl, KCl, CaCl_2_, FeCl_3_, AA, and UA due to the inclusion of PVP and PVA as a protective layer. Compared to other advanced systems like PdNPs-GNPs/MWCNTs (with a lower LOD of 0.008 μM but lower sensitivity of 83.00 μA mM^–1^ cm^–2^), and AgNPs/CuO/ITO (with an excellent LOD of 0.0517 μM but a limited linear range up to 0.50 mM), the Ag–Cu/MWCNTs electrode offers a balanced performance, delivering a wide detection range, acceptable sensitivity, and enhanced anti-interference capacity, essential for practical bioanalytical applications.

**Table 1:** Comparison of Glucose Sensors Based on Different Electrode Materials.^a^,^b^

Electrode	Sensitivity (μA mM^–1^ cm^–2^)	LOD (μM)	Linear range (mM)	Interferents	Protective layer	References
**Ag–Cu/NF**	7745.70	0.0800	0.0050–3.50	AA, UA	–	^52^
**AgNPs/CuO/ITO**	1347.00	0.0517	0.0005–0.50	AA, UA, DA, NaCl	–	^76^
**Pd/MWCNTs/parafilm**	1275.00	0.2000	1.0000–22.0	AA, UA, DA	–	^77^
**Cu–Cu**_**2**_ **O NPs@3DG/GCE**	230.89	16.000	0.8000–10.0	AA, UA, DA, APAP	–	^78^
**Ag/Cu**_**2**_ **O/AZO/ITO glass**	221.60	–	3.3300–11.1	AA, UA, DA, NaCl	–	^79^
**Au/ZrO**_**2**_ **@MWCNTs/GCE**	162.52	0.9500	0.0100–13.0	AA, UA, DA, NaCl	–	^80^
**Cu/Glass**	145.52	2.8700	0.0100–0.20	AA, UA	–	^81^
**PdNPs-GNPs/MWCNTs/GCE**	83.00	0.0080	0.0250–10.0	AA, UA, DA, NaCl	–	^82^
**AgNW/NiCo LDH/GCE**	71.420	0.6600	0.0020–6.0	AA, UA, DA	–	^83^
**Ag–Cu/MWCNTs/GCE**	237.20	1.2000	0.5000–10.0	AA, UA, NaCl, KCl, CaCl_2_, FeCl_3_	PVP+PVA	This work

a The table summarizes key performance parameters, including sensitivity, LOD (limit of detection), linear range, interferents, and protective layer for various nanomaterial-modified electrodes reported in recent literature, highlighting the advantages of hydrogel-protected Ag–Cu/MWCNTs composite in terms of electrocatalytic activity and anti-interference performance.

b Note: AA: ascorbic acid; UA: uric acid; DA: dopamine; APAP: acetaminophen.

#### 3.4.4 Amperometric Investigation

The amperometric performance of the electrodes was further assessed in 0.1 M NaOH solution under continuous stirring by comparing Acid–MWCNTs, Ag–MWCNTs, Cu–MWCNTs, and Ag_5_–Cu_5_/MWCNTs. As shown in Figure 5A, Acid–MWCNTs exhibited a minimal current response to glucose addition. This is attributed to the noncatalytic nature of MWCNTs, where glucose undergoes self-oxidation to gluconolactone in the presence of OH^–^, but the lack of metal-based catalytic sites limits the current enhancement. In contrast, the Ag–MWCNTs electrode (Figure 5B) showed improved electrocatalytic activity due to the presence of silver. However, the current response was lower and less stable than expected, likely due to Ag nanoparticle aggregation and surface cracking induced by drying stress, which reduces the effective catalytic area.^84^ For the Cu–MWCNTs electrode (Figure 5C), although the current was slightly lower than that of the Ag–MWCNTs, it exhibited good stability. This is likely due to the uniform distribution of Cu and the absence of noticeable surface degradation. The Ag_5_–Cu_5_/MWCNTs electrode (Figure 5D) demonstrated the highest and most stable current responses among all samples. This synergistic enhancement arises from the combined catalytic effects of Ag and Cu. Moreover, the presence of Cu helps suppress Ag aggregation and mitigates stress-induced surface damage, leading to improved current stability and sensitivity.^85,86^

**Figure 5:**
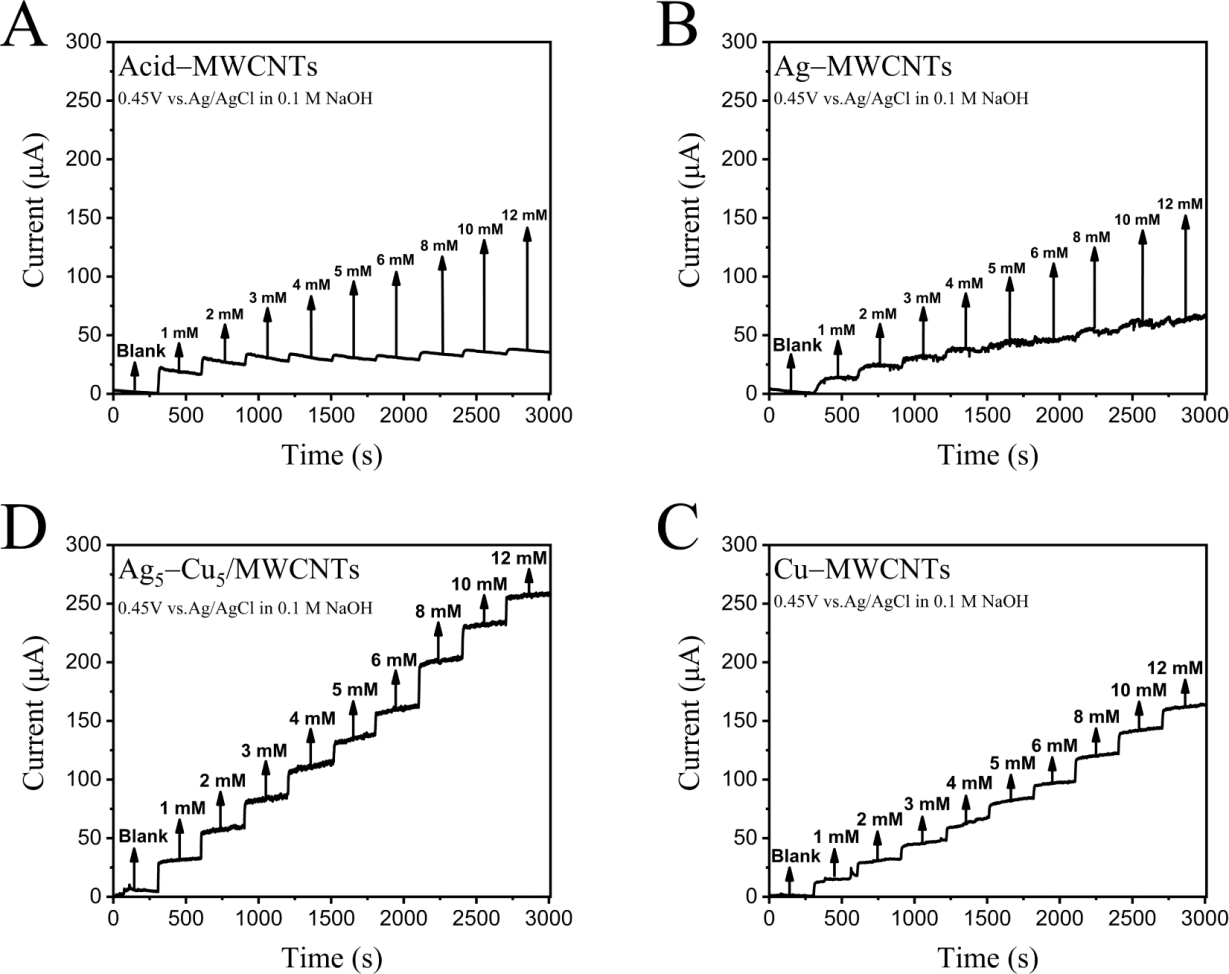
Amperometric responses of different modified electrodes toward successive additions of glucose (1–12 mM) at 0.45 V vs Ag/AgCl in 0.1 M NaOH: (A) Acid-treated MWCNTs (Acid-MWCNTs), (B) Ag/MWCNTs, (C) Cu/MWCNTs, and (D) Ag_5_–Cu_5_/MWCNTs. All electrodes show stepwise increases in current with increasing glucose concentration, with the Ag_5_–Cu_5_/MWCNTs demonstrating the highest and most stable current response, indicating superior electrocatalytic activity for glucose oxidation.

#### 3.4.5 Effect of Interfering Species on Sensing Response

One of the most important problems for the nonenzymatic sensors is to reduce the effect of endogenous interfering species like sodium chloride (NaCl), uric acid (UA), ascorbic acid (AA), and so on.^87–89^ These species coexist along with glucose in the physiological fluid of the human body. The normal blood glucose level of a healthy human is 3–8 mM, which is almost ten times higher than other species such as NaCl (0.1 mM), UA (0.1 mM), and AA (0.1 mM) present in a blood sample. Although the electron transfer rates of these substances are high and their concentrations are very low compared to glucose, the oxidation currents generated by these substances may become interference currents of glucose and affect the calculated blood sugar values.^90^

To assess the anti-interference performance of the developed electrodes, amperometric measurements were carried out at a fixed potential of 0.45 V vs Ag/AgCl under continuous stirring. Electrodes based on Acid–MWCNTs, Ag–MWCNTs, Cu–MWCNTs, and Ag_5_–Cu_5_/MWCNTs were sequentially exposed to glucose and various common interfering species, including NaCl, KCl, CaCl_2_, FeCl_3_, AA, and UA (Figure 6). As shown in Figure 6A, the Acid–MWCNTs exhibited a noticeable current increase upon the addition of CaCl_2_, likely due to coordination interactions between Ca^2+^ ions and MWCNT surfaces.^91^ In the case of Ag/MWCNTs (Figure 6B), a gradual decline in current was observed over time, reflecting glucose consumption and surface passivation due to Ag aggregation. Additional glucose additions failed to restore the current, suggesting active site saturation. Cu/MWCNTs (Figure 6C) displayed relatively stable currents, with slower current decay than Ag/MWCNTs, indicating a better retention of catalytic activity. Notably, the Ag_5_–Cu_5_/MWCNTs electrode (Figure 6D) exhibited the highest and most stable current responses among all samples, confirming the synergistic effects of Ag and Cu.

**Figure 6:**
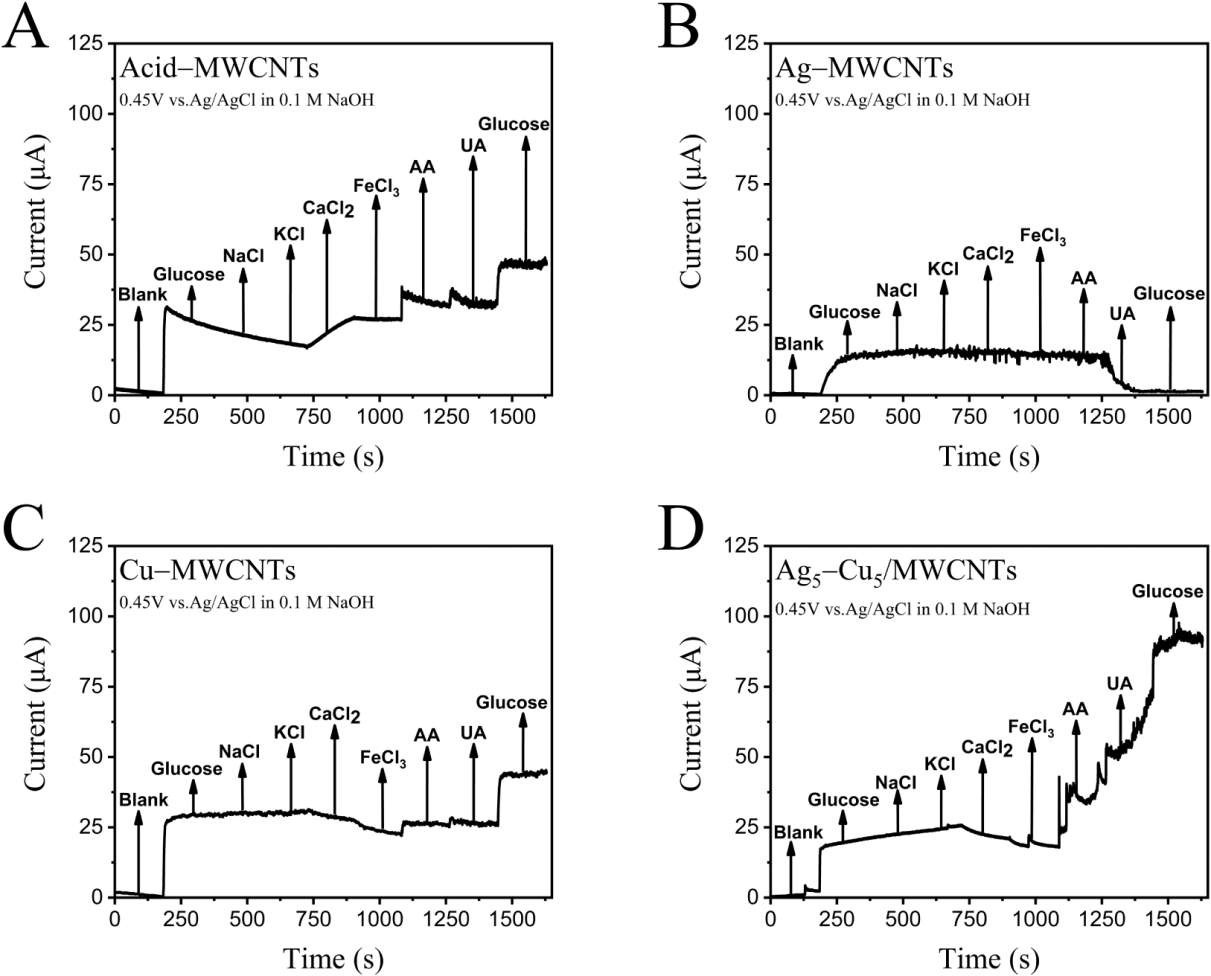
Amperometric responses of different modified electrodes to glucose (initial and final additions) and various potential interfering substances, measured at 0.45 V vs Ag/AgCl in 0.1 M NaOH: (A) Acid-MWCNTs, (B) Ag/MWCNTs, (C) Cu/MWCNTs, and (D) Ag_5_–Cu_5_/MWCNTs. Each test included successive additions of glucose, NaCl, KCl, CaCl_2_, FeCl_2_, ascorbic acid (AA), and uric acid (UA). Among the tested electrodes, Ag_5_–Cu_5_/MWCNTs demonstrate the highest selectivity, showing a strong current response to glucose with minimal interference from common species (chlorides). Notably, fluctuations in current are observed upon the addition of AA and UA, indicating partial interference.

Among the interferents, AA and UA produced the most significant fluctuations in current, underscoring their strong interference potential. Therefore, these two substances were chosen as representative interferents in subsequent studies involving protective layers of PVP and PVA for anti-interference enhancement. In the amperometric method without a protective layer (Figure 7A), the current gradually increased after the addition of AA and UA, even though glucose was not added, indicating that the current response was primarily induced by these interferents. After the introduction of a mixed PVP/PVA protective layer, the current rise due to AA and UA was significantly suppressed, though some fluctuations remained (Figure 7B–E). As the concentration of the protective layer increased, these fluctuations were not substantially minimized across the 0.3–10 wt % range. However, at a protective layer concentration of 30 wt %, a marked improvement in interference suppression was observed. In Figure 7F, it can be seen that when GCE uses a 30 wt % protective layer, the current only increases with the little increase in glucose concentration, so the protective layer does not directly increase the current. In Figure S2, there is no significant difference in the current increase between 10, 3, and 0.3 wt % protection layers, but the current increases significantly after introducing a 30 wt % protection layer. According to the work by the Schuhmann group,^92^ interference from negatively charged species such as uric acid (UA) and ascorbic acid (AA) can be effectively suppressed through electrostatic repulsion provided by a negatively charged polymer layer. This electrostatic effect, however, has a minimal influence on neutral glucose molecules. Furthermore, the protective polymer layer exhibits excellent hydrophilicity, which facilitates the formation of a hydration layer and enhances the membrane swelling. These properties allow glucose molecules to diffuse rapidly into the sensing layer. In our study, the PVA/PVP protective layer consists of negatively charged polymers. As such, interference from UA and AA can be mitigated through electrostatic repulsion. Meanwhile, the hydrophilic nature of the PVA/PVP layer promotes glucose diffusion, thereby improving interaction with the Ag_5_–Cu_5_/MWCNTs electrode.

**Figure 7:**
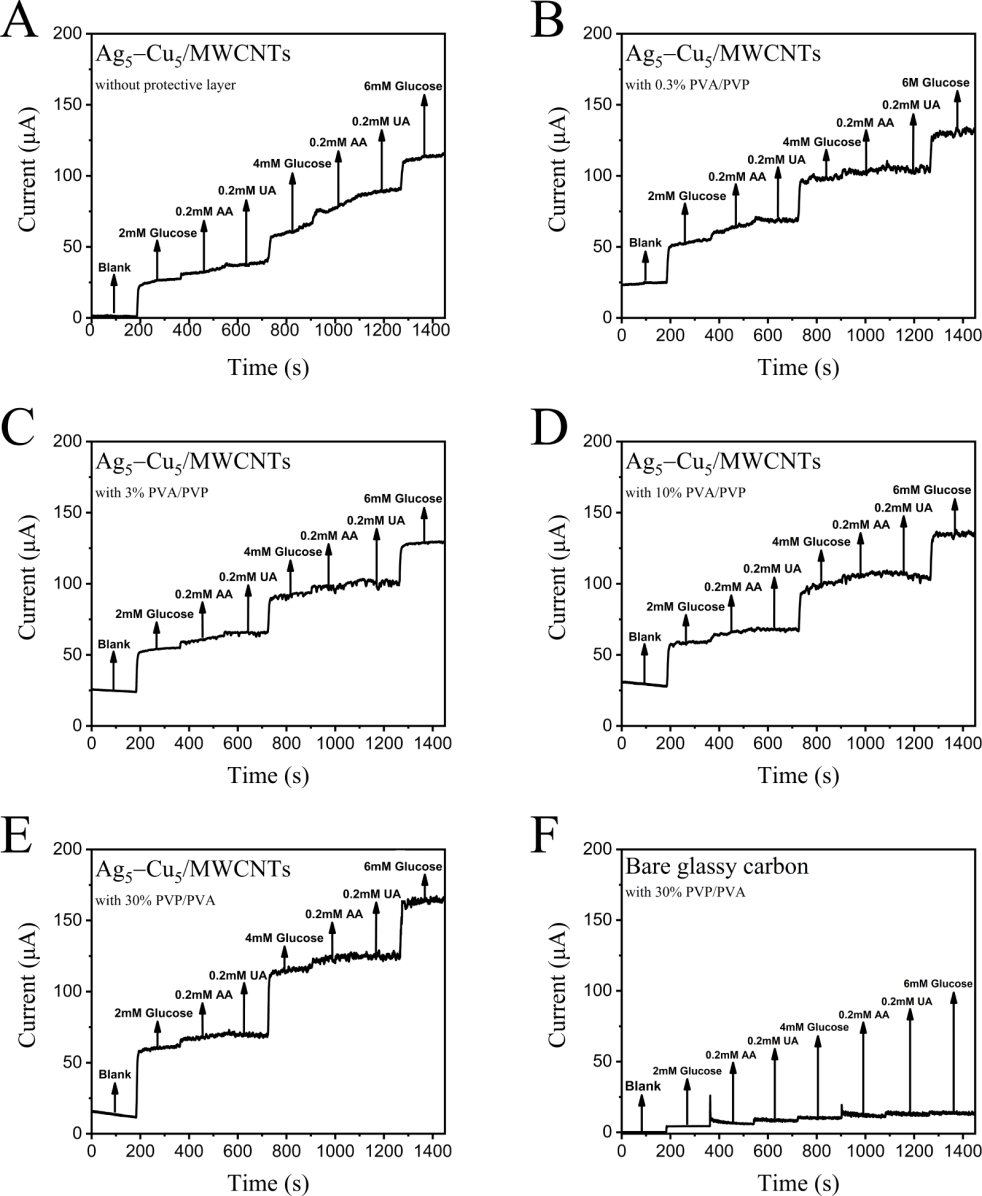
Amperometric responses to successive additions of glucose (2, 4, and 6 mM) in the presence of common interfering species (0.2 mM ascorbic acid (AA) and 0.2 mM uric acid (UA)), using electrodes coated with different concentrations of PVA/PVP protective layers: (A) Ag_5_–Cu_5_/MWCNTs without a protective layer, (B) Ag_5_–Cu_5_/MWCNTs with 0.3% PVA/PVP, (C) Ag_5_–Cu_5_/MWCNTs with 3% PVA/PVP, (D) Ag_5_–Cu_5_/MWCNTs with 10% PVA/PVP, (E) Ag_5_–Cu_5_/MWCNTs with 30% PVA/PVP, and (F) Bare GCE with 30% PVA/PVP (control). All electrodes were tested at 0.45 V vs Ag/AgCl in 0.1 M NaOH. As the PVA/PVP concentration increases, the influence of interfering species (AA and UA) on the current response is progressively suppressed, while the glucose response remains stable. The 30% protective layer shows the most effective discrimination against AA and UA, confirming its excellent anti-interference capability.

## 4 Conclusions

In this study, a nonenzymatic glucose sensor based on Ag–Cu/MWCNTs nanocomposites was prepared via a hydrothermal method. The optimal mole ratio of Ag:Cu at 1:1 demonstrated superior electrocatalytic activity, attributed to the synergistic interaction between Ag and Cu nanoparticles and the high conductivity of MWCNTs. Structural and electrochemical characterizations confirmed the uniform distribution and high surface area of the nanocomposites, which significantly enhanced glucose oxidation under alkaline conditions. The sensor exhibited a wide linear detection range from 0.5 to 10 mM with a good correlation coefficient of *R* ^2^ = 0.99697, a sensitivity of 237.2 μA·mM^–1^·cm^–2^, and a detection limit of 1.2 μM. To improve selectivity and minimize interference from endogenous substances such as uric acid (UA) and ascorbic acid (AA), a PVP/PVA hydrogel film was applied as a molecular sieve. The interference from UA and AA can be mitigated through electrostatic repulsion. Furthermore, the PVP/PVA layer absorbs water and swells to form water-filled channels that allow neutral glucose molecules to diffuse readily. This selective permeability enhances the sensor’s selectivity and measurement accuracy. Overall, the integration of Ag–Cu/MWCNTs with this selective polymer coating offers a promising route for the development of highly sensitive, stable, and selective nonenzymatic glucose sensors suitable for real-world biomedical applications, including glucose detection in blood and sweat.

## Supplementary Material



## Data Availability

Data used is available throughout the manuscript text.

## Notes

Electron microscopy image and corresponding energy-dispersive X-ray spectrum of Ag–Cu nanoparticles on MWCNTs; amperometric glucose sensing calibration curves with different hydrogel protective layer concentrations, and additional experimental details (PDF)
