# Supplementary material for: Systematic Nonenzymatic Glucose Detection Using Hydrogel-Protected Ag–Cu/MWCNTs Nanocomposites
Source: ACS Omega. 2025 Oct 10;10(41):48844–57. doi: 10.1021/acsomega.5c06932 (PMC12547558; doi:10.1021/acsomega.5c06932)
Supplement: Supplementary file 1 [file ao5c06932_si_001.pdf]

Supporting Information for

Systematic Non-enzymatic Glucose Detection Using Hydrogel-protected Ag–Cu/MWCNTs Nanocomposites

Jian-Kai Huang[#], Sin-Yu Chen[#], and Meng-Chang Lin[*]

Department of Materials Science and Engineering, National Chung Hsing University, 40227 Taichung, Taiwan, ROC

[#]The authors have equal contributions to this article

[*]Corresponding author: E-mail: mengchanglin@dragon.nchu.edu.tw

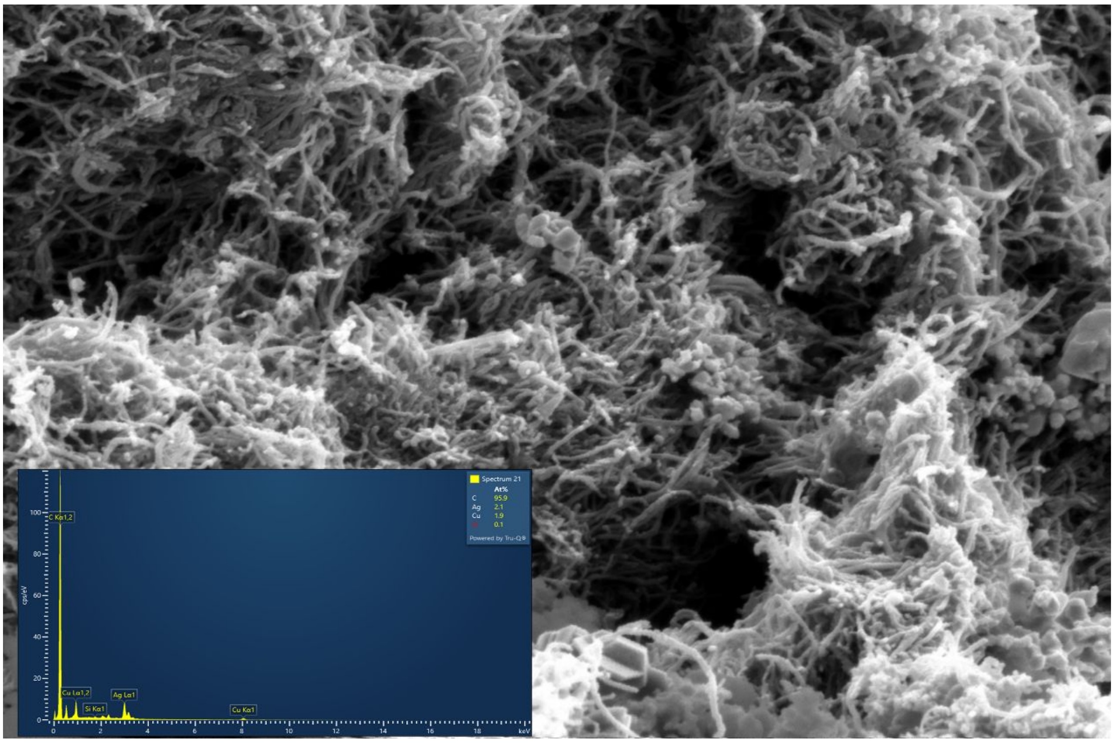

Figure S1. The scanning electron microscopy (SEM) image shows the morphology and dispersion of Ag–Cu nanoparticles on the MWCNTs surface. The corresponding energy-dispersive X-ray (EDX) spectrum confirms the presence and atomic composition of Ag and Cu, indicating successful alloy formation and uniform distribution across the MWCNTs framework.

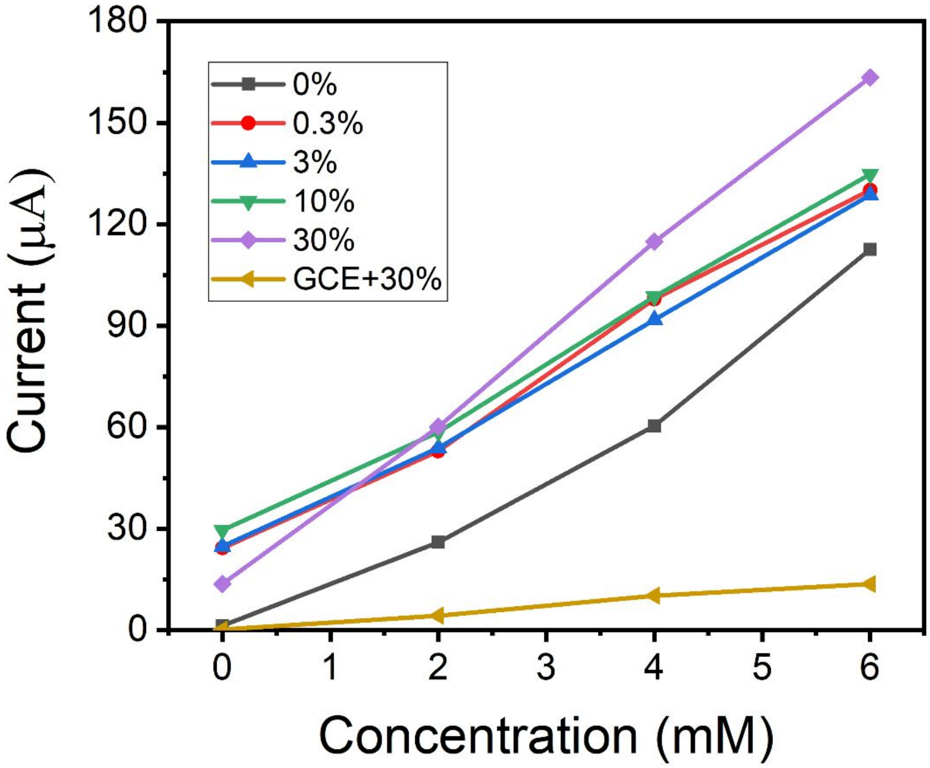

Figure S2. Amperometric glucose sensing calibration curves with different PVA/PVP protective layer concentrations. Comparison of current responses versus glucose concentration (0-6 mM) for electrodes coated with 0.3 wt.%, 3 wt.%, 10 wt.%, and 30 wt.% protective hydrogel layers.
